# Panel Work in Theory and Practice

**Published:** 1914-01-10

**Authors:** 


### The National Medical Union.

### Panel Work in Theory and in Practice.

Barely a month ago the extravagant overstatements current in the Ministerial party press as to the remuneration of panel doctors were mentioned in these columns; and the prophecy was ventured that the next step would be a demand for curtailing the pay of those who attend insured persons under the Act (The Hospital, December 13, 1913, p. 298). It was scarcely expected that this demand would be expressed so very speedily; but those—whether they be for or against the present system of remuneration for medical services under the Act—who may have been sceptical a month ago would do well to read a leading article in the *Daily News* for December 31, one of the most authoritative exponents of Governmental and Socialist policy.

"That the payment under the Act is excessive for the work done is no longer disputable. The arrangement is, of course, a provisional one, and the authorities are doubtless engaged on a drastic revision of the scheme." So says the *Daily News*, and if this outspoken intimation does not rouse the medical profession, we do not know what will. The article goes on to demand a State Medical Service as the "only practicable way out of the present unsatisfactory position."

Incidentally, this last admission throws light on the sincerity of the Radical Party and its press organs, which have never ceased protesting for the last twelve months how extraordinarily successful everything connected with the working of National Insurance has been from first to last. A confession that the position is "unsatisfactory" means a good deal from such a quarter; for nothing would have induced assent to such a proposition from any supporter of the Government a short time ago.

### Drastic Revision.

Official information is regularly available to the chief newspapers on the Ministerial side before it becomes public property through recognised channels. So we may take it that when the *Daily News* speaks of "drastic revision" of the terms for panel doctoring, there is at least a possibility that the matter has been discussed in responsible quarters. Once more, panellers will please note the prospect thus opened up for them. They can scarcely be expected to agree that their rewards are excessive; not, at least, those who have the serious intention of doing their duty honestly by their patients, as most of them doubtless have. But the *Daily News* says that it is "no longer disputable," and that settles it. Whether this semi-official pronouncement is a kite of the Chancellor's, flown to test the winds of medical opinion, or represents a Socialist point of view more advanced than any as yet adopted by the Government as a whole, is immaterial. What does matter is that the medical profession—or rather, its panelite section—has had the gage of battle plainly thrown down to it. The struggle which is impending will be, it is now evident, not a fight to secure better terms under the Act, but to maintain intact those which have been already granted. Defence, not offence, is the *rôle* that is to be thrust upon the doctors; and they will do well to remember that the best defence is usually a vigorous offensive. It is hard to imagine any more cogent reason for preparation and organisation. Forewarned is forearmed; but only when the warning is heeded and the armoury is filled up. If the National Medical Union had wanted any *raison d'être*, the *Daily News* would have supplied one.

### The Truth about Panel Practice.

When the panel system was first put before the medical profession, and men were invited to join it, one of the inducements held out was that on the average about 25 per cent. of the insured would need medical attendance during any one year. It was, presumably, upon the basis of friendly society experience that this estimate was made, as was the actuarial calculation of the amount of sick benefit claims that might be expected. The latter was proved to be woefully wrong; and actual experience is showing the fallacy of the former estimate also.

Let us take a few figures which show how much work the panel doctor does for his money—that payment which is so excessive, according to the editor of the *Daily News*. A correspondent supplies us with the results of his own panel practice, which are full of instruction in this connection. As we shall presently show, his experience is probably not in the least exceptional; indeed, it is probable that many panel practitioners do even more work for their pay than our correspondent does.

From April 15, 1913, to December 31, 1913, the total number of panel patients allotted to him was 1,245. It does not necessarily follow that he received 1,245 capitation fees in full each quarter, as some of the insured may have been on his lists for broken periods alone. The total number who have received treatment in the eight and a half months is 708, a good deal more than half the total insured persons. These 708 have been attended in all 4,115 times: 3,505 surgery attendances and 610 home visits.

It is a fair assumption that in the period January to April the sickness prevailing among the insured will be at least as great as in the period October to December. On that assumption the total attendances on 1,245 insured persons during the full year will work out at nearly 6,000, including some 930 visits, or thereabouts. In other words, each panel patient receives 4.8 attendances a year on the average; for which the doctor is paid 7s., or more probably rather less; this works out at less than 1s. 5d. each. What extravagant remuneration for a man to receive whose education has cost him six or seven or more years and some hundreds of pounds! That he is at work all day and every day, at all hours, in all weathers, should appeal to the sympathies of the *Daily News*, ever sympathetic to claims for shorter working hours. But, no; a drastic revision of these rates of pay is imminent we are told.

It would not admittedly be safe or fair to found an argument on one single case such as that just quoted. Nor would it have been done had there been any reason to suppose the figures exceptional or unusual. On the contrary, our correspondent's letter contains evidence tending to show that his practice among the insured has not been unusually onerous. For he mentions that of the 708 who sought his treatment, 407 were males and 301 were females. It is a commonplace that female insured constitute proportionately by far the major part of a panel doctor's patients. So it is certain (the actual figures are not given in the letter) that of the 1,245 on the list the great bulk must be males. It follows that a list of the same size, but preponderatingly feminine in sex, would provide for a panel doctor a very great deal more than the 6,000 attendances mentioned in this case, and a correspondingly reduced rate of remuneration.

### Medical Aid Institutes.

A fortnight ago attention was drawn (The Hospital, December 27, p. 349) to the iniquitous system of sweating perpetrated in certain parts of Wales by working men's "medical aid institutes" with the connivance of the Insurance Commissioners. England also harbours a few of these institutes; and it is pleasing to note that in England, as in Wales, there is a widespread refusal on the part of hospital staffs to have anything to do with the medical officers of such "institutes," or with their patients except in cases of urgency. The Gloucester Infirmary, for instance, has taken this line with the Forest of Dean Medical Aid Association; and is threatened in consequence with the loss of some subscriptions from working-class organisations in the Forest. However, the loss of the subscriptions will not cripple the infirmary to any great degree. For at a recent quarterly meeting of the governors the Chairman (Canon Eyre) pointed out that during the past year in-patients alone from the Forest cost the hospital £1,167, without reckoning in some 658 out-patients from the same district; whereas the contributions from the same district are always under £400 a year, and in 1913 were not much more than half that sum. The meeting expressed approval of the stand which the medical staff are making in this matter. An encouraging sign!

One other journalistic lucubration of the week deserves mention. This is the astonishing advice tendered to the medical profession by the *Manchester Guardian* on January 2—not to insist on the right of non-panel doctors' certificates being accepted as evidence of sickness by the approved societies. This legal right was established months ago, in the teeth of fierce opposition, through litigation which was undertaken by the National Medical Guild. The reason which the editor of the *Guardian* gives for the abandonment of this hardly won privilege is very curious. It would be a grave error in tactics, he says, to insist on the right, because it is always a mistake to drive one's adversary to desperation. Who the adversary may be he does not explain; it may be the panel doctor, the approved society, the insured person, or the Commissioners. How or why any of these should be driven to desperation by non-panel doctors' certificates is almost equally mysterious. The *Guardian* does, it is true, suggest that non-panel doctors will give certificates indiscriminately; but this is also freely alleged against panellers. It might reasonably be argued that non-panel doctors, as a class, are more likely to take sufficient care over diagnosis and treatment and less likely to give mistaken certificates, than those on the panel. In any case, the solicitude for the medical profession is very touching; but it looks uncommonly like one of those Greek gifts which wise men mistrust.

### The Serfdom of the Panel Doctor.

Every day brings evidence of the restrictions being gradually imposed upon the panel doctor by the National Insurance Commissioners, and in greater and more humiliating degree by the local Insurance Committees. It is pleasant, however, to be able to record an exception from the North. At the December monthly meeting of the Kirkcaldy Burgh Insurance Committee, permission was graciously accorded, but not without a lively discussion and in the face of considerable opposition, to the panel doctors of Kirkcaldy to be relieved of the consulting hour on Wednesday evenings. But the dispensation was not obtained without a sacrifice, for the Medical Committee are reported to have offered to give an extra half-hour each on Monday and Thursday evenings. The "evening off" was approved by twenty-six votes to five.

